# 

**Published:** 1969-10

**Authors:** 


### UNIVERSITY OF BRISTOL

### *Faculty of Medicine*

THE University grants the degrees of Bachelor of Medicine and Surgery (M.B., Ch.B.), Master of Surgery (Ch.M.). Doctor of Medicine (M.D.), Bachelor of Dental Surgery (B.D.S.), Master of Dental Surgery (M.D.S.), and Bachelor of Veterinary Science (B.V.Sc.), as well as diplomas in Public Health (D.P.H.), and in Dental Surgery (L.D.S.).

Clinical instruction is provided in the United Bristol Hospitals and in some of the Hospitals in the Bristol area under the control of the South Western Regional Hospital Board. Mechanical and clinical instruction in Dentistry is carried out in the University's Dental Hospital and School.

*INCLUSIVE FEES*

| | |
|---|---|
| For the M.B., Ch.B. curriculum . | £82 per annum |
| For the B.D.S. curriculum . . . | £82 per annum |
| For the L.D.S. curriculum . . . | £82 per annum |
| For the B.V.Sc. curriculum . . . | £82 per annum |

---

*For additional particulars apply to*

*THE REGISTRAR, THE UNIVERSITY, SENATE HOUSE, BRISTOL 2*

THE

BRISTOL MEDICO-CHIRURGICAL JOURNAL

*(Incorporating the Medical Journal of the South-West)*

Established 1883

Vol. 84 (iv) October 1969 No. 312

*PUBLISHED QUARTERLY*

*ANNUAL SUBSCRIPTION* **16/-** *POST FREE*

### BRISTOL MEDICO-CHIRURGICAL SOCIETY

President 1968/69 : Mr. A. L. Eyre-Brook

President elect : Dr. F. J. W. Lewis

Honorary Secretary : Dr. I. S. Bailey, 7 Percival Road, Bristol 8.

Honorary Treasurer : Dr. J. Macrae, Ham Green Hospital, Pill.

The Society was founded in 1874. In 1883 publication of this Journal began, and has continued quarterly since then. During the years 1949 to 1962 it appeared under the title of "The Medical Journal of the South West".

The Society founded a medical library in 1890, which in 1892 was combined with that of the Bristol Medical School. This became the University of Bristol Medical Library in 1925, and an agreement in that year confirmed the privilege of Members to use the University Medical Library.

### THE BRISTOL MEDICO-CHIRURGICAL JOURNAL

*Editorial Committee*

*Editors*

Dr. N. J. Brown, Southmead Hospital, Bristol.
Dr. A. B. Raper, Royal Infirmary, Bristol.

*Members*

Mr. H. B. Griffith

Dr. M. B. Lennard

Mr. J. B. M. Roberts

Professor R. C. Wofinden

Dr. D. W. Wright

*Secretary*

Mr. A. E. S. Roberts, The Library, Medical School, University Walk, Bristol, 8.

The Hon. Treasurer and Hon. Secretary of the Society.

### NOTICE TO CONTRIBUTORS

The Journal is especially concerned to provide a place for the recording of medical thought, interests, and practice in and around Bristol. It therefore welcomes articles that, as well as being of immediate interest to members, will document the local and contemporary medical scene for our successors.

Original articles are invited provided that they have not been published elsewhere. References in the text should be by author's name, with year of publication in parenthesis; they should be listed at the end in alphabetical order. Illustrations should be supplied ready for reproduction. Line drawings should be in indian ink on firm white paper or board. The author will be informed if it is found necessary to ask him to pay for the making of blocks.

The following contributions will be especially welcome : notes on forthcoming events, meetings held, degrees conferred, staff appointments, honours and public appointments and other local news.

Printed by
F. Bailey & Son Dursley, Glos.

